# The Book World of Medicine and Science

**Published:** 1895-09-14

**Authors:** 


### THE BOOK WORLD OF MEDICINE AND SCIENCE.

WALKS IN BELGIUM. CYCLING, DRIVING, BY RAIL, AND ON FOOT. Edited by PERCY LINDLEY. With new maps and illustrations, and a Chapter on the French Ardennes. (Price 6d.)

This little book is a very pleasing specimen of its class. Well got up, clearly and concisely written, it may be commended warmly to all who seek an inexpensive pleasure trip in the Ardennes. It contains excellent maps, as well as woodcut illustrations, and the various routes by which the tourist may travel from one place to another are indicated in the text, together with the chief features of interest passed by on the way. The editor is indeed to be congratulated on having produced such an excellent little work, and at so low a price that it would be a cheap book even if it only contained half its 94 pages.

THE STORY OF PRIMITIVE MAN. EDWARD CLODD. (London: George Newnes, Limited. 1895.)

As we are reminded in this book, it was only quite a short while ago that our school histories of Britain began with the invasion of Julius Cæsar. About man and his doings prior to this event such histories were silent. Indeed, little more than a century back Boswell reports Johnson as saying: "All that is really *known* of the ancient state of Britain is contained in a few pages. We *can* know no more than what the old writers have told us." And yet, as Mr. Clodd explains, even at the time that Johnson was speaking of, there was lying in the Sloane Museum a rudely-chipped flint weapon which had been found at the end of the seventeenth century, associated with an elephant's tooth, "opposite to black Marys, near Grayes Inn Lane," in which street Johnson then lived. But years passed before it was known that these and other unheeded relics epitomised the early history of man, and the condition of the Thames Valley when he and a strange group of animals lived there in a dim and dateless past. It is from the knowledge gained from such significant facts and from relics of man's presence that much has been gathered in recent years about the blood relationship of various races, and, more than this, about man's place in the long chain of life on the globe. Mr. Clodd has given us in his little book an admirable sketch of what the science of anthropology has done in revealing the history of primitive man; no book of the same compass has done this before. Anthropology is a branch of knowledge which has made more rapid advance during the past fifty years than any other dealing with the byegone history of mankind.

The plan adopted by the writer in the present instance in illustrating the progress of his science is through dividing stage of civilisation into the Stone Age, the Bronze Age, and the Iron, a classification now generally adopted, but which had long ago been anticipated by certain ancient writers, notably by Lucretius in the poem, "De Rerum Natura." "Arms of old were hands, nails, and teeth, and stones, and boughs broken off from the forests, and flames and fire, as soon as they had become known. Afterwards the force of iron and copper was discovered; and the use of copper was known before that of iron, as its nature is easier to work, and it is found in greater quantity." And though no really definite dates can be given to these several divisions, the one thing certain is the succession of the stages of culture which they denote.

This little volume is profusely illustrated, and we commend it to the reading and intelligent public as possessing an interest at once fascinating and absorbing.

MYXŒDEMA AND THE THYROID GLAND. By JOHN D. GIMLETTE, M.R.C.S. (London: J. and A. Churchill. 1895. Pp. 128.)

Mr. Gimlette's work is an amplification of his inaugural thesis for the Portuguese title of Medico-cirurgião. It forms a very reliable and convenient epitome of the present state of knowledge of the subject in all its aspects, including the history, ætiology, pathology, and treatment of the affection. The value of the work would have been greatly enhanced if references to the authors quoted had been appended, as was done by Dr. Marius Wilson in his little book on Myxœdema.*

* "Myxœdema and the Effects of Climate on the Disease." (Scientific Press. 1894.)

